# Relationship between stress hyperglycaemic ratio and incidence of in-hospital cardiac arrest in patients with acute coronary syndrome: a retrospective cohort study

**DOI:** 10.1186/s12933-024-02128-y

**Published:** 2024-02-09

**Authors:** Kui Li, Xueyuan Yang, Yunhang Li, Guanxue Xu, Yi Ma

**Affiliations:** Department of Cardiovascular Medicine, Affiliated Hospital of Zunyi Medical University, No. 149 Dalian Road, Zunyi, 563099 Guizhou China

**Keywords:** Stress hyperglycaemic ratio, Incidence, In-hospital cardiac arrest patients, Acute coronary syndrome, Retrospective cohort study

## Abstract

**Background:**

The stress hyperglycaemic ratio (SHR), a new marker that reflects the true hyperglycaemic state of patients with acute coronary syndrome (ACS), is strongly associated with adverse clinical outcomes in these patients. Studies on the relationship between the SHR and in-hospital cardiac arrest (IHCA) incidence are limited. This study elucidated the relationship between the SHR and incidence of IHCA in patients with ACS.

**Methods:**

In total, 1,939 patients with ACS who underwent percutaneous coronary intervention (PCI) at the Affiliated Hospital of Zunyi Medical University were included. They were divided into three groups according to the SHR: group T1 (SHR ≤ 0.838, N = 646), group T2 (0.838< SHR ≤ 1.140, N = 646), and group T3 (SHR3 > 1.140, N = 647). The primary endpoint was IHCA incidence.

**Results:**

The overall IHCA incidence was 4.1% (N = 80). After adjusting for covariates, SHR was significantly associated with IHCA incidence in patients with ACS who underwent PCI (odds ratio [OR] =  2.6800; 95% confidence interval [CI] =  1.6200–4.4300; *p*<0.001), and compared with the T1 group, the T3 group had an increased IHCA risk (OR =  2.1800; 95% CI =  1.2100–3.9300; *p* =  0.0090). In subgroup analyses, after adjusting for covariates, patients with ST-segment elevation myocardial infarction (STEMI) (OR =  3.0700; 95% CI =  1.4100–6.6600; *p* =  0.0050) and non-STEMI (NSTEMI) (OR =  2.9900; 95% CI =  1.1000–8.1100; *p* =  0.0310) were at an increased IHCA risk. After adjusting for covariates, IHCA risk was higher in patients with diabetes mellitus (DM) (OR =  2.5900; 95% CI =  1.4200–4.7300; *p* =  0.0020) and those without DM (non-DM) (OR =  3.3000; 95% CI =  1.2700–8.5800; *p* =  0.0140); patients with DM in the T3 group had an increased IHCA risk compared with those in the T1 group (OR =  2.4200; 95% CI =  1.0800–5.4300; *p* =  0.0320). The restriction cubic spline (RCS) analyses revealed a dose-response relationship between IHCA incidence and SHR, with an increased IHCA risk when SHR was higher than 1.773. Adding SHR to the baseline risk model improved the predictive value of IHCA in patients with ACS treated with PCI (net reclassification improvement [NRI]: 0.0734 [0.0058–0.1409], *p* =  0.0332; integrated discrimination improvement [IDI]: 0.0218 [0.0063–0.0374], *p* =  0.0060).

**Conclusions:**

In patients with ACS treated with PCI, the SHR was significantly associated with the incidence of IHCA. The SHR may be a useful predictor of the incidence of IHCA in patients with ACS. The addition of the SHR to the baseline risk model had an incremental effect on the predictive value of IHCA in patients with ACS treated with PCI.

## Background

Stress-induced hyperglycaemia (SIH), a transient elevation of blood glucose associated with disease stress, is independently associated with poor short- and long-term clinical outcomes in patients with acute coronary syndrome (ACS) [1–3]. However, this correlation is stronger in patients without diabetes mellitus (DM) than in those with DM [2], suggesting that acutely elevated glucose levels, rather than chronically elevated glucose levels, may be causative in terms of a worse prognosis in patients with ACS. Most previous studies have used the admission blood glucose level (ABG) to determine SIH; however, a combination of an acute hyperglycaemic state and chronic blood glucose level determines the ABG. Thus, in patients with DM combined with ACS, an elevated ABG does not fully reflect the degree of acute hyperglycaemia. Considering this, to reflect the true acute hyperglycaemic state and to better assess the actual glycaemic status of patients, Robert et al. proposed a new relative hyperglycaemic index (stress hyperglycaemic ratio [SHR]), defined as the ABG divided by the chronic blood glucose level calculated using glycated haemoglobin (HbA1c). The authors reported that the SHR is a more effective predictor of poor prognosis in critically ill patients than absolute hyperglycaemia[4]. Several studies have reported that the SHR is significantly associated with adverse clinical outcomes in patients with ACS [5–13].

Reportedly, the SHR shows better predictive value than does ABG in patients with ACS [7, 9, 10]. However, there are limited studies on the relationship between the SHR and incidence of in-hospital cardiac arrest (IHCA). This study aimed to elucidate the relationship between the SHR and incidence of IHCA in patients with ACS.

## Methods

### Study design and population

**Fig. 1:**
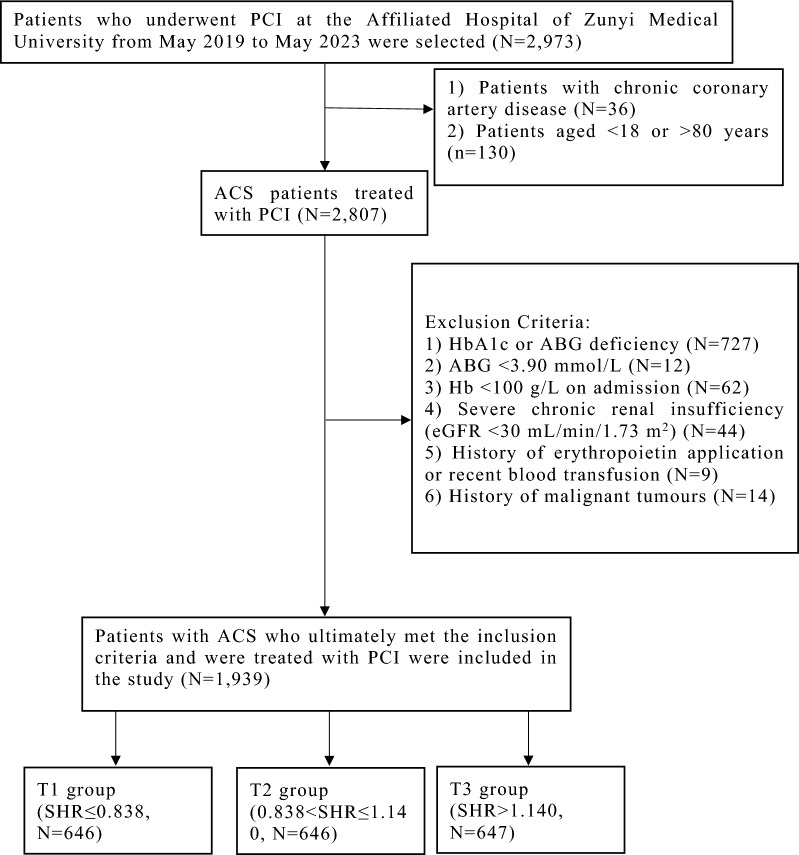
Patient inclusion flowchart

This retrospective cohort study was conducted at the Affiliated Hospital of Zunyi Medical University. The study was conducted in accordance with the tenets of the Declaration of Helsinki and was authorised by the Ethics Committee of the Affiliated Hospital of Zunyi Medical University. Written informed consent was obtained from all patients. Patients who underwent percutaneous coronary intervention (PCI) at the Affiliated Hospital of Zunyi Medical University between 1 May 2019 and 1 May 2023 were included in this study. Patients who met the following criteria were included: (1) those aged 18–80 years and (2) those with ACS treated with PCI. Patients meeting the following criteria were excluded: (1) no HbA1c or ABG data; (2) ABG level < 3.90 mmol/L; (3) haemoglobin level < 100 g/L on admission; (4) severe chronic renal insufficiency (estimated glomerular filtration rate [eGFR] < 30 mL/min/1.73 m^2^); (5) history of erythropoietin application or recent blood transfusion; or (6) history of malignancy (Fig. 1). Ultimately, the data of 1,939 patients with ACS treated with PCI were included in the final analysis. Patients were categorised into three groups based on the SHR: group T1 (SHR ≤ 0.838, N = 646), group T2 (0.838 < SHR ≤ 1.140, N = 646), and group T3 (SHR > 1.140, N = 647). The primary endpoint was the incidence of IHCA.

### Data measurement and definitions

The baseline demographic and clinical data of all patients were retrospectively collected from the medical records of Zunyi Medical University Hospital. Demographic data included the patients' age, sex, body mass index, smoking status, comorbidities (hypertension, dyslipidaemia, DM), previous stroke, family history, history of myocardial infarction(MI), previous PCI, and previous coronary artery bypass grafting. Clinical data included the patients' systolic blood pressure and diastolic blood pressure at admission, primary diagnosis at admission (ST-segment elevation myocardial infarction [STEMI], non-STEMI [NSTEMI], unstable angina pectoris [UA]), imaging and surgical data (transradial approach, left main stem disease, left anterior descending branch disease, left circumflex branch disease, right coronary artery disease, coronary chronic total occlusion lesion, number of diseased vessels, bifurcation lesion, number of stents, length of stents, diameter of stents, thrombolytic therapy, drug-coated balloon, and transluminal coronary rotational atherectomy [rotablator]), laboratory tests and findings (left ventricular ejection fraction, triglycerides [TG], total cholesterol, high-density lipoprotein cholesterol, low-density lipoprotein cholesterol, ABG, HbA1c, haemoglobin, creatinine [Cr], uric acid, ultrasensitive C-reactive protein), and the patient's medication regimen during hospitalisation (insulin, oral hypoglycaemic drugs, aspirin, P2Y12 inhibitors, statins, β-blockers, angiotensin-converting enzyme inhibitors/angiotensin receptor II antagonist). IHCA was defined as chest compressions and/or defibrillation performed on hospitalised patients [14]. ABG was defined as randomised blood glucose measured for the first time within 24 h of admission. Blood glucose was measured with an AU5800 system (Beckman Coulter, California, USA), and HbA1c was measured using high-performance liquid chromatography (D10, BIO-RAD, California, USA). Body mass index was calculated as body weight (Kg) divided by the square of the height (m). The eGFR was calculated according to the MDRD formula [15]: male: eGFR = 186 × Cr^−1.154^ × age^−0.203^, female: eGFR = 186 × Cr^−1.154^ × age^−0.203^ × 0.742. The estimated chronic glucose level was calculated using the following formula: (28.7 × HbA1c%) − 46.7) [16]. The SHR was defined as the ABG divided by the estimated chronic glucose level [4], and the SHR was calculated according to the following formula: SHR = ABG/[(28.7 × HbA1c [%]) − 46.7]. Coronary artery disease was defined as ≥ 50% luminal narrowing of at least one major coronary artery (left anterior descending, left circumflex, or right coronary artery). Left main stem lesions were defined as ≥ 50% left main coronary artery stenosis. DM was defined as a history of type 2 DM or an HbA1c level ≥ 6.5%, whereas non-DM was defined as an HbA1c level < 6.5% [17]. Stroke was defined as a history of cerebral haemorrhage, ischemic stroke, or transient ischemic attack.

### Statistical analyses

Continuous variables with a normal distribution are presented as mean ± standard deviation, and continuous variables with a non-normal distribution are presented as median (interquartile range). Categorical variables are presented as numbers (percentages). Comparisons between three groups of continuous variables that were normally distributed and variance-aligned were analysed using analysis of variance, and comparisons between three groups of continuous variables that were not normally distributed or variance-aligned were analysed using the Kruskal–Wallis rank-sum test. Comparisons between three groups of categorical variables were performed using the chi-square test. Logistic regression analysis was performed, providing the odds ratio (OR) and 95% confidence interval (CI) to explore the association between the SHR and IHCA incidence. In the current study, model 1 was not adjusted; model 2 was adjusted for clinically relevant baseline variables or baseline variables screened from a pool of variables using least absolute shrinkage and selection operator regression. Variables for inclusion were carefully chosen, given the number of events available, to ensure parsimony of the final model. The following covariates were adjusted for age, smoking, SBP, DBP, number of diseased vessels, CTO disease, thrombolytic therapy, eGFR, high-sensitivity C-reactive protein (hs-CRP), LVEF, and LDL-C. Restricted cubic spline (RCS) analysis explored the relationship between the SHR and IHCA incidence. In addition, considering that the correlation between the SHR and IHCA was approximately linear below and above the SHR value corresponding to an OR equal to 1, a linear model was used to calculate the OR for each increase in the standard deviation of the SHR. Diagnostic value analyses were performed using receiver operating characteristic (ROC) curves, and the area under the curve (AUC), measured using the C-statistic, was calculated to quantify the predictive ability of the logistic model for IHCA. AUC comparisons between models were assessed using DeLong's test. In addition, the Net Reclassification Index (NRI) and Integrated Discriminant Improvement Index (IDI) were calculated to further assess the additional predictive value of the SHR for IHCA beyond the identified risk factors. Finally, subgroup analyses based on primary diagnosis and DM status at admission were performed using logistic regression analysis. A *p*-value of < 0.05 was considered statistically significant, and all analyses were performed using a two-sided approach. All statistical analysis were performed using R version 4.2.3.

## Results

### Baseline characteristics

A total of 1,939 patients with ACS treated with PCI were included in this study. The mean age of the patients was 60 (53, 69) years. There were 1,427 (73.6%) male patients, 1,138 (58.7%) patients with DM, 454 (23.4%) patients with STEMI, 454 (23.4%) patients with NSTEMI, and 1,031 (53.2%) patients with UA. There were significant differences in previous coronary artery bypass grafting, DM, diagnosis on admission, trans-radial approach, coronary chronic total occlusion lesions, left ventricular ejection fraction, TG level, ABG level, HbA1c, SHR, insulin use, oral hypoglycaemic drug use, P2Y12 inhibitors use, and angiotensin-converting enzyme inhibitor/angiotensin receptor II antagonist use among the three groups (all *p* < 0.05) (Table 1). Patients in the T3 group were more likely to have a higher TG and ABG level, and the proportion of patients with DM and insulin use was significantly higher than in the other groups.

**Table 1:** Demographic and clinical baseline data for the three groups

	Total	T1 (SHR ≤ 0.838)	T2 (0.838< SHR ≤ 1.140)	T3 (SHR3 > 1.140)	
	N = 1939	N = 646	N = 646	N = 647	*p*
Age (years)	60(53,69)	60(53,70)	60(52,68)	62 (53,70)	0.114
Male	1,427 (73.6)	493 (76.3)	469 (72.6)	465 (71.9)	0.151
BMI (kg/m^2^)	23.88 (22.04, 26.44)	23.88 (21.88, 26.11)	23.86 (21.99, 26.33)	24.22 (22.05, 26.64)	0.267
SBP (mmHg)	123 (111, 136)	124 (112, 135)	122 (111, 136)	122 (111, 136)	0.661
DBP (mmHg)	75 (68, 84)	76 (68, 85)	75 (67, 84)	75 (68, 84)	0.438
Smoking					0.890
Current	816 (42.1)	262 (40.6)	279 (43.2)	275 (42.5)	
Former	282 (14.5)	99 (15.3)	91 (14.1)	92 (14.2)	
Never	841 (43.4)	285 (44.1)	276 (42.7)	280 (43.3)	
Previous stroke	222 (11.4)	72 (11.1)	73 (11.3)	77 (11.9)	0.903
Family history	11 (0.6)	3(0.5)	3(0.5)	5 (0.8)	0.695
Previous MI	794 (40.9)	283 (43.8)	254 (39.3)	257 (39.7)	0.192
Previous PCI	414 (21.4)	155 (24.0)	124 (19.2)	135 (20.9)	0.102
Previous CABG	14 (0.7)	10 (1.5)	2 (0.3)	2 (0.3)	0.010
Hypertension grade					0.588
1	116 (6.0)	38 (5.9)	39 (6.0)	39 (6.0)	
2	320 (16.5)	113 (17.5)	114 (17.6)	93 (14.4)	
3	726 (37.4)	250 (38.7)	233 (36.1)	243 (37.6)	
Dyslipidaemia	855 (44.1)	266 (41.2)	288 (44.6)	301 (46.5)	0.147
DM	1,138 (58.7)	378(58.5)	335(51.9)	425 (65.7)	< 0.001
Diagnosis on admission					< 0.001
STEMI	454 (23.4)	113 (17.5)	158 (24.5)	183 (28.3)	
NSTEMI	454 (23.4)	154 (23.8)	156 (24.1)	144 (22.3)	
UA	1,031 (53.2)	379 (58.7)	332 (51.4)	320 (49.5)	
Transradial approach	1,838 (94.8)	623 (96.4)	615 (95.2)	600 (92.7)	0.010
LM disease	103 (5.3)	41 (6.3)	38 (5.9)	24 (3.7)	0.078
LAD disease	1,262 (65.1)	401(62.1)	428 (66.3)	433 (66.9)	0.140
LCX disease	604 (31.2)	203 (31.4)	194 (30.0)	207 (32.0)	0.735
RCA disease	724 (37.3)	236 (36.5)	260 (40.2)	228 (35.2)	0.155
CTO disease	1,018 (52.5)	304 (47.1)	355 (55.0)	359 (55.5)	0.003
Number of diseased vessels					0.418
1	946 (48.8)	326 (50.5)	316 (48.9)	304 (47.0)	
2	619 (31.9)	198 (30.7)	197 (30.5)	224 (34.6)	
3	374 (19.3)	122 (18.9)	133 (20.6)	119 (18.4)	
Bifurcation lesion	25 (1.3)	8(1.2)	8(1.2)	9 (1.4)	0.961
Number of stents	2(1,3)	2(1,3)	2(1,3)	2(1,2)	0.235
Length of stents (mm)	46 (28, 72)	48 (29, 74)	45.5 (28, 73)	45 (27, 69)	0.379
Diameter of stents (mm)	3 (2.75, 3.50)	3 (2.81, 3.50)	3 (2.75, 3.50)	3 (2.75, 3.38)	0.139
Thrombolytic therapy	43 (2.2)	11 (1.7)	13 (2.0)	19 (2.9)	0.293
Drug-coated balloon	120 (6.2)	48 (7.4)	35 (5.4)	37 (5.7)	0.270
Rotablator	34 (1.8)	16 (2.5)	9 (1.4)	9 (1.4)	0.230
LVEF (%)	56 (46.00, 61.00)	57 (47.25, 61.00)	55 (45.00, 60.00)	56 (46.50, 60.00)	0.033
TG (mmol/L)	1.86 (1.26, 2.92)	1.66 (1.15, 2.51)	1.88 (1.27, 2.94)	2.12 (1.39,3.33)	< 0.001
TC (mmol/L)	4.79 (3.89, 5.70)	4.67 (3.82, 5.62)	4.83 (3.97,5.78)	4.82 (3.90, 5.71)	0.096
HDL-C (mmol/L)	1.08 (0.92, 1.25)	1.07 (0.93, 1.25)	1.09 (0.94, 1.26)	1.07 (0.90, 1.25)	0.129
LDL-C (mmol/L)	2.90 (2.29, 3.57)	2.84 (2.22, 3.54)	2.95 (2.33, 3.63)	2.93 (2.31, 3.56)	0.125
ABG (mmol/L)	7.75 (5.89, 11.46)	5.59 (4.82, 6.91)	7.30 (6.22, 9.68)	12.04 (9.33, 16.29)	< 0.001
HbA1c (%)	6.6 (5.8, 8.2)	6.7 (5.9, 8.2)	6.2 (5.7, 7.9)	6.8 (5.8, 8.3)	< 0.001
SHR	0.97 (0.78, 1.24)	0.72 (0.63, 0.78)	0.97 (0.90, 1.05)	1.40 (1.24, 1.61)	< 0.001
Hb (g/L)	140.0 (129.0, 152.0)	139.5 (128.0, 151.0)	140.0 (129.0, 152.0)	140.0 (128.5, 152.0)	0.273
Cr (µmol/L)	77 (65.5, 92.0)	77 (66.0, 92.0)	75 (65.0, 90.0)	78 (66.0, 94.5)	0.188
eGFR (mL/min/1.73 m^2^)	89.61 (72.65, 106.14)	90.20 (73.10, 105.89)	91.21 (75.44, 106.72)	87.14 (70.92, 105.43)	0.074
Uric acid (µmol/L)	354.00 (295.00, 429.00)	352.00 (300.00, 424.75)	358.50 (288.00, 433.75)	350.00 (298.00, 427.00)	0.664
hs-CRP (mg/L)	3.50 (1.20, 12.38)	3.50 (1.18,12.08)	3.45 (1.19, 11.92)	3.65 (1.24, 14.30)	0.572
Insulin	476 (24.5)	130 (20.1)	136 (21.1)	210 (32.5)	< 0.001
Oral hypoglycaemic drugs	897 (46.3)	307 (47.5)	265 (41.0)	325 (50.2)	0.003
Aspirin	1,906 (98.3)	635 (98.3)	638 (98.8)	633 (97.8)	0.437
P2Y12 inhibitors	1,920 (99.0)	645 (99.8)	635 (98.3)	640 (98.9)	0.018
Statins	1,921 (99.1)	643 (99.5)	640 (99.1)	638 (98.6)	0.221
β-blockers	1,599 (82.5)	530 (82.0)	527 (81.6)	542 (83.8)	0.551
ACEIs/ARBs	1,529 (78.9)	524 (81.1)	518 (80.2)	487 (75.3)	0.022

Data are presented as means ± SDs, medians (interquartile ranges), or n (%)

Stress Hyperglycaemic Ratio, SHR; Body Mass Index, BMI; Systolic Blood Pressure, SBP; Diastolic Blood Pressure, DBP; Diabetes Mellitus, DM; Myocardial Infarction, MI; Percutaneous Coronary Intervention, PCI; Coronary Artery Bypass Grafting, CABG; Left Anterior Descending, LAD; Left Circumflex, LCX; Right Coronary Artery, RCA; Chronic Total Occlusion, CTO; ST-Elevation Myocardial Infarction, STEMI; Non-ST-Elevation Myocardial Infarction, NSTEMI; Unstable Angina, UA; High-Density Lipoprotein Cholesterol, HDL-C; Low-Density Lipoprotein Cholesterol, LDL-C; Triglycerides, TG; Total Cholesterol, TC; Admission Blood Glucose, ABG; Haemoglobin A1c, HbA1c; Left Ventricular Ejection Fraction, LVEF; Estimated Glomerular Filtration Rate, eGFR; High-Sensitivity C-Reactive Protein, hs-CRP; Angiotensin-Converting Enzyme Inhibitor/Angiotensin Receptor Blocker, ACEI/ARB

### Clinical outcomes

**Fig. 2:**
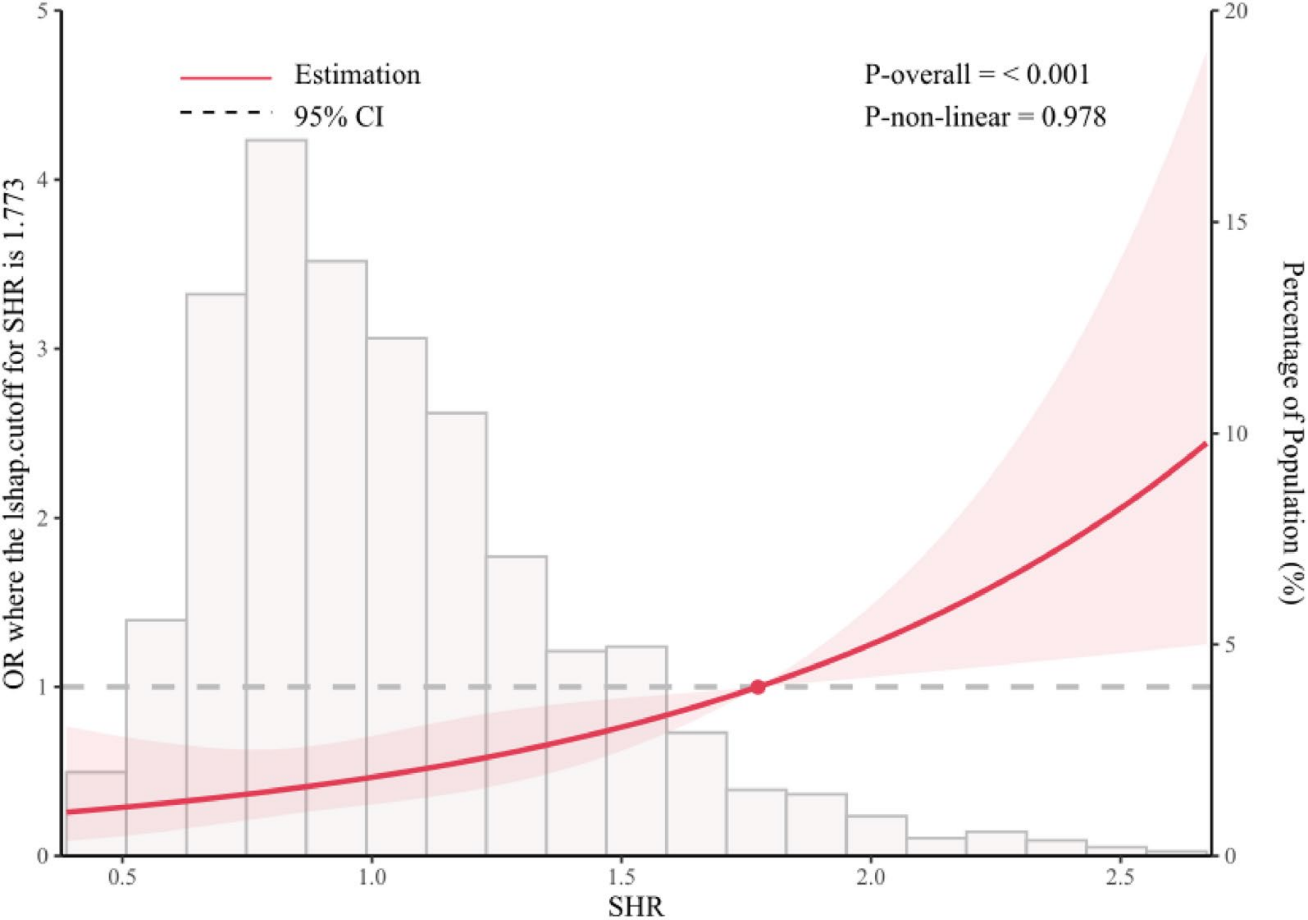
Relationship between stress hyperglycaemic ratio and incidence of in-hospital cardiac arrest in patients with ACS. Only 95% of the data is displayed. Odds ratios are indicated by solid lines and 95% CIs by shaded areas. acute coronary syndrome, ACS

The overall IHCA incidence was 4.1% (N = 80), and the difference in IHCA incidence among the three groups was statistically significant (*p* < 0.001) (Table 2). In model 1, SHR was significantly associated with the risk of developing IHCA (OR = 3.0300; 95% CI = 1.9500–4.6800; *p* < 0.001) (Table 3). After adjusting for potential risk factors in model 2, SHR was an independent risk factor for IHCA in patients with ACS (OR  =  2.6800; 95% CI = 1.6200–4.4300; *p* < 0.001). In model 1, the incidence of IHCA was 2.61 times higher in the T3 group than in the T1 group (OR = 2.6100; 95% CI = 1.4900–4.5600; *p* < 0.001). In model 2, the incidence of IHCA was 2.18 times higher in the T3 group than in the T1 group (OR = 2.1800; 95% CI=1.2100–3.9300; *p* = 0.0090). The results of the RCS analysis showed a dose-response relationship between SHR and IHCA incidence even after adjusting for confounders in model 2 (nonlinear *p*-value = 0.978) (Fig. 2).When the SHR was < 1.773, the OR for IHCA incidence slowly changed. When the SHR was > 1.773, the OR of IHCA incidence increased significantly. The OR per standard deviation(SD) for predicting IHCA increased to 1.3924 (1.0873–1.7844) when the SHR was < 1.773, and increased to  1.9020 (0.8373–4.5468) when the SHR was > 1.773 (Table 4).

**Table 2:** Incidence of in-hospital cardiac arrest in the three groups

Endpoint	Total (N = 1939)	T1 (N = 646)	T2 (N = 646)	T3 (N = 647)	*p*
IHCA	80 (4.1%)	18 (2.8%)	17 (2.6%)	45 (7.0%)	< 0.001

**Table 3:** Relationship between the stress hyperglycaemic ratio and incidence of in-hospital cardiac arrest

	Events/N	Model 1			Model 2		
		OR	95% CI	*p*	OR	95% CI	*p*
SHR	80/1,939	3.0300	1.9500-4.6800	< 0.001	2.6800	1.6200-4.4300	< 0.001
T1	18/646	Reference			Reference		
T2	17/646	0.9400	0.4800–1.8500	0.8640	0.8100	0.4000–1.6300	0.5560
T3	45/647	2.6100	1.4900–4.5600	< 0.001	2.1800	1.2100–3.9300	0.0090
*p* for trend				< 0.001			0.0033

Odds Ratio, OR; Confidence Interval, CI; Stress Hyperglycaemic Ratio, SHR; Group with SHR ≤ 0.838, T1; Group with 0.838 < SHR ≤ 1.140, T2; Group with SHR > 1.140, T3; *p*-value, p

**Table 4:** Relationship between SHR (per 1 SD) and in-hospital cardiac arrest incidence in PCI treated ACS patients

	OR per SD	95% CI
SHR ≥ 1.773	1.9020	0.8373–4.5468
SHR < 1.773	1.3924	1.08730–1.7844

Odds Ratio, OR; Confidence Interval, CI; Stress Hyperglycaemic Ratio, SHR; Standard Deviation, SD; PCI Percutaneous Coronary Intervention; ACS, Acute Coronary Syndrome

### Subgroup analyses

Subgroup analyses of the correlation between the SHR and IHCA in different populations were performed according to the primary diagnosis at admission (STEMI, NSTEMI, UA) and DM status (DM, non-DM).

Table 5 shows the relationship between the SHR and incidence of IHCA in patients with STEMI, NSTEMI, and UA. The study showed that there was no interaction of SHR with IHCA among the STEMI, NSTEMI, and UA subgroups (*p* = 0.5918). In STEMI patients, both model 1 (OR = 3.2700; 95% CI = 1.6800–6.3700; *p* < 0.001) and model 2 (OR = 3.0700; 95% CI = 1.4100–6.6600; *p* = 0.0050) showed that the SHR was significantly associated with the risk of developing IHCA. In patients with NSTEMI, model 1 showed that the SHR was significantly associated with the risk of IHCA (OR = 3.2300; 95% CI = 1.4100–7.4300; *p*=0.0060) and that the incidence of IHCA in the T3 group was 2.63 times higher than that in the T1 group (OR = 2.6300; 95% CI = 1.0500–6.5800; *p* = 0.0400);model 2 also showed that the SHR was significantly associated with the risk of developing IHCA (OR = 2.9900; 95% CI = 1.1000–8.1100; *p* = 0.0310).

**Table 5:** Relationship between the stress hyperglycaemia ratio and incidence of in-hospital cardiac arrest in patients with ST-segment elevation myocardial infarction, patients with non-ST-segment elevation myocardial infarction, and patients with unstable angina pectoris

Diagnosis on admission	Events/N	Model 1			Model 2		
		OR	95% CI	*p*	OR	95% CI	*p*
STEMI	36/454	3.2700	1.6800–6.3700	< 0.001	3.0700	1.4100–6.6600	0.0050
T1	6/113	Reference			Reference		
T2	9/158	1.0800	0.3700–3.1200	0.8910	0.8700	0.2900–2.6500	0.8050
T3	21/183	2.3100	0.9000–5.9200	0.0800	1.9500	0.7300–5.2000	0.1800
NSTEMI	28/454	3.2300	1.4100–7.4300	0.0060	2.9900	1.1000–8.1100	0.0310
T1	7/154	Reference			Reference		
T2	5/156	0.7000	0.2200–2.2400	0.5430	0.6100	0.1700–2.2300	0.4530
T3	16/144	2.6300	1.0500–6.5800	0.0400	2.7000	0.9700–7.5600	0.0580
UA	16/1,031	1.9500	0.6900–5.5500	0.2090	1.5100	0.4900–4.7000	0.4740
T1	5/379	Reference			Reference		
T2	3/332	0.6800	0.1600–2.8800	0.6020	0.6700	0.1500–3.0200	0.5990
T3	8/320	1.9200	0.6200–5.9200	0.2580	1.5200	0.4400–5.2100	0.5040

ST-Elevation Myocardial Infarction, STEMI; Non-ST-Elevation Myocardial Infarction, NSTEMI; Unstable Angina, UA; Stress Hyperglycaemic Ratio, SHR; Group with the Lowest SHR Value (reference group), T1; Group with Intermediate SHR Values, T2; Group with the Highest SHR Value, T3; Odds Ratio, OR; Confidence Interval, CI; *p*-value, *p*

Table 6 shows the relationship between the SHR and incidence of IHCA in patients with and without DM. The study showed that there was no interaction of the SHR with IHCA between the DM and non-DM subgroups (*p* = 0.7211). In patients with DM, model 1 showed that the SHR was significantly associated with the risk of developing IHCA (OR = 2.9300; 95% CI = 1.7400–4.9300; *p* < 0.001) and that the incidence of IHCA was 2.67 times higher in the T3 group than in the T1 group (OR = 2.6700; 95% CI = 1.2400–5.7800; *p* = 0.0120); model 2 also showed that the SHR was significantly associated with the risk of IHCA (OR = 2.5900; 95% CI = 1.4200–4.7300; *p* = 0.0020) and that the incidence of IHCA in the T3 group was 2.42 times higher than that in the T1 group (OR = 2.4200; 95% CI = 1.0800–5.4300; *p* = 0.0320). In non-DM patients, model 1 showed that the SHR was significantly associated with the risk of developing IHCA (OR = 3.5200; 95% CI = 1.5100–8.1900; *p* = 0.0040) and that the incidence of IHCA in the T3 group was 2.69 times higher than that in the T1 group (OR = 2.69; 95% CI = 1.1900–6.0800; *p* = 0.0170); model 2 also showed that the SHR was significantly associated with the risk of developing IHCA (OR = 3.3000; 95% CI = 1.2700–8.5800; *p* = 0.0140).

**Table 6:** Relationship between the stress hyperglycaemia ratio and incidence of in-hospital cardiac arrest in non-diabetes patients and diabetes patients

Diabetes status	Events/N	Model 1			Model 2		
		OR	95% CI	*p*	OR	95% CI	*p*
Non-DM							
T1	9/268	Reference			Reference		
T2	5/311	0.4700	0.1600–1.4200	0.1810	0.3500	0.1000–1.1800	0.0900
T3	19/222	2.6900	1.1900–6.0800	0.0170	2.4100	0.9500–6.0800	0.0630
DM							
T1	9/378	Reference			Reference		
T2	12/335	1.5200	0.6300–3.6600	0.3470	1.3900	0.5600–3.4600	0.4840
T3	26/425	2.6700	1.2400–5.7800	0.0120	2.4200	1.0800–5.4300	0.0320

Diabetes Mellitus, DM; T1; Group with Intermediate SHR Values, T2; Group with the Highest SHR Value, T3; Odds Ratio, OR; Confidence Interval, CI; *p*-value, *p*

### Incremental effect of SHR on predicting IHCA

**Fig. 3:**
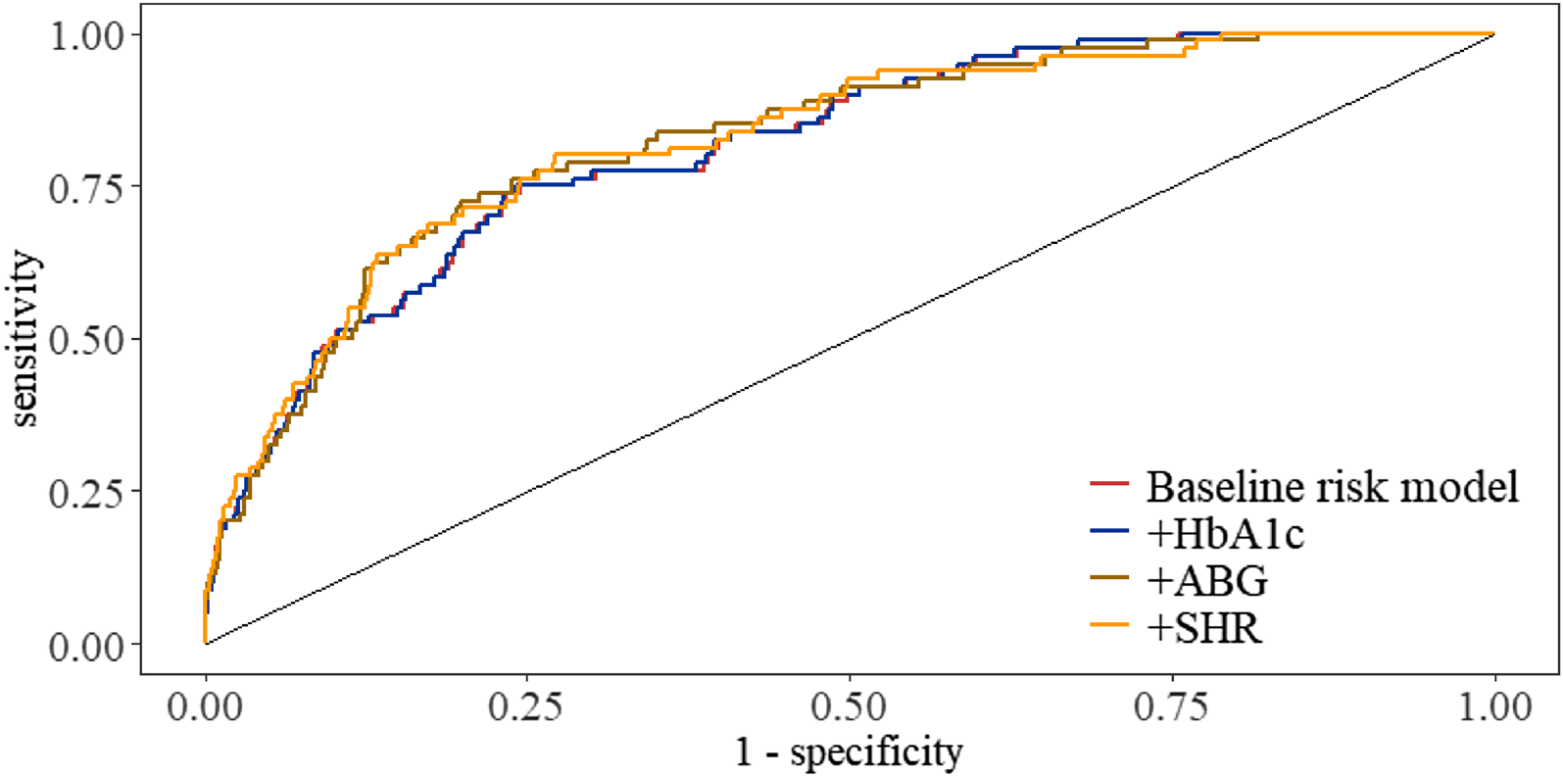
Receiver operating characteristic curves assessing the predictive ability of HbA1c, ABG, and SHR for IHCA. Baseline risk model vs +HbA1c in ACS patients treated with PCI, baseline risk model vs +ABG in ACS patients treated with PCI, baseline risk model vs +SHR in ACS patients treated with PCI. Baseline risk model includes age, smoking, SBP, DBP, number of diseased vessels, CTO disease, thrombolytic therapy, eGFR, hs-CRP, LVEF, and LDL-C

In the analysis of patients with ACS treated with PCI, ROC curves were constructed to assess the predictive ability of the baseline risk model ( baseline risk model includes age, smoking, SBP, DBP, number of diseased vessels, CTO disease, thrombolytic therapy, eGFR, hs-CRP, LVEF, and LDL-C) and baseline risk model plus HbA1c, ABG, and the SHR for IHCA, respectively (Fig. 3). The C-statistic, NRI, and IDI are presented in Table 7. The results of the study showed a significant incremental effect of SHR on the predictive value of the baseline risk model in patients with ACS treated with PCI   (NRI: 0.0734 [0.0058–0.1409], *p* = 0.0332; IDI: 0.0218 [0.0063–0.0374], *p* = 0.0060).

**Table 7:** Incremental predictive value and predictive power of various models with NRI, IDI, and C-statistics

Model	C-statistic (95% Cl)	*p*	NRI (95% Cl)	*p*	IDI (95% Cl)	*p*
Baseline risk model	0.8123 (0.7677–0.8568)	Ref.	Ref.		Ref.	
+ HbA1c	0.8125 (0.7680–0.8570)	0.4489	0.0000 (-0.0021–0.0021)	1.0000	0.0001 (-0.0003–0.0001)	0.4018
+ ABG	0.8237 (0.7793–0.8680)	0.1419	0.0353 (-0.0191–0.0898)	0.2032	0.0115 (-0.0011–0.0241)	0.0731
+ SHR	0.8242 (0.7786–0.8698)	0.1941	0.0734 (0.0058–0.1409)	0.0332	0.0218 (0.0063–0.0374)	0.0060

Baseline risk model includes age, smoking, SBP, DBP, number of diseased vessels, CTO disease, thrombolytic therapy, eGFR, hs-CRP, LVEF, and LDL-C.

Admission Blood Glucose, ABG; Confidence Interval, CI; Integrated Discrimination Improvement, IDI; Net Reclassification Improvement, NRI; References, ref; Stress Hyperglycaemic Ratio, SHR

## Discussion

In this study, the SHR was significantly associated with the incidence of IHCA in patients with ACS treated with PCI. The SHR showed a dose–response relationship with the incidence of IHCA. The addition of the SHR to the baseline risk model had an incremental effect on the predictive value of IHCA in patients with ACS treated with PCI.

SIH in patients with ACS may result from pancreatic β-cell dysfunction and insulin resistance [18, 19]. Reportedly, pancreatic β-cell dysfunction affects insulin release [20, 21], thereby increasing glucagon and glucose levels in patients with ACS [22, 23]. In addition, dysregulation of the sympathetic nervous system and renin–angiotensin–aldosterone system in the context of ACS leads to increased stimulation of adrenergic and angiotensin II receptors and the occurrence of insulin resistance [24]. ABG refers to the random blood glucose level measured within the first 24 h after a patient's admission to the hospital [2, 25]. Prior research has characterised SIH using ABG [2, 26–31], wherein SIH is described as a transient elevation of blood glucose linked to the stress experienced during the disease and identified as an independent factor associated with unfavourable short- and long-term clinical outcomes in patients diagnosed with ACS [1–3]. Hyperglycaemia exerts direct deleterious effects on the ischemic myocardium through various mechanisms, including oxidative stress, inflammation, apoptosis, endothelial dysfunction, hypercoagulability, platelet aggregation, and impairment of ischemic preadaptation [32–35]. However, it remains controversial whether an elevated ABG level (SIH) is merely a manifestation of severe disease or is associated with serious consequences such as complications or death [36–38]. High values of ABG do not necessarily indicate elevated blood glucose levels after an acute myocardial infarction (AMI), especially in patients with DM accompanied by chronic elevated blood glucose levels [4]. Therefore, Robert et al. [4] proposed the SHR, which can distinguish whether ABG levels represent acute or chronic glucose elevation, defined as the ratio of ABG to chronic glucose levels, and found that the SHR is a better biomarker of critical illness than absolute hyperglycaemia. The formula [28.7 × HbA1c (%) − 46.7] can determine chronic blood glucose levels to gain new insights into the relationship between blood glucose and poor prognosis by correcting blood glucose levels for HbA1c [16, 39].

Several studies have examined the predictive value of SHR for a short-term adverse prognosis in patients with ACS [9, 11–13]. Xu et al. [11] enrolled 7,476 patients with STEMI with the primary endpoints of major adverse cardiovascular events (MACEs) and all-cause mortality with a follow-up period of 30 days, and showed that the SHR was independently associated with the risk of developing MACEs and mortality. Chen et al. [12] recruited 341 consecutive patients aged ≥75 years with a diagnosis of AMI, and the study endpoints were in-hospital all-cause mortality and in-hospital major adverse cardiovascular and cerebrovascular events (MACCE), suggesting that the SHR may serve as a simple and independent indicator of poor prognosis during hospitalisation in patients with AMI without diabetes. Marenzi et al. [13] recruited 474 patients with DM combined with AMI, with the primary endpoint of acute kidney injury, and found that the SHR was a better predictor of the occurrence of acute kidney injury than was the ABG level. Elsewhere, Marenzi et al. [9] recruited 1,553 patients with AMI, with the primary endpoints of in-hospital mortality, acute pulmonary oedema, and cardiogenic shock. The SHR was a better predictor of morbidity and mortality during hospitalisation than was the ABG level. Other studies have examined the predictive value of the SHR for long-term adverse prognosis in patients with ACS [5–8]. Yang et al. [5] recruited 5,562 consecutive patients with ACS treated with PCI, with a primary endpoint of MACC and a 2-year follow-up period. During a median follow-up period of 28.3 months, they found that the SHR correlated in a U-shape with 2-year MACCE and MACE rates, and in a J-shape with in-hospital cardiac mortality and MI. Cui et al. [6] demonstrated a significant positive correlation between SHR and long-term mortality in patients with AMI with and without DM by conducting a prospective, multicentre study of 6,892 patients with AMI with the primary endpoint of 2-year all-cause mortality. Sia et al. [7] recruited 5,841 patients with STEMI and 4,105 with NSTEMI. The study endpoint was all-cause mortality with a follow-up period of 1 year, revealing that the SHR was the most consistent independent predictor of 1-year all-cause mortality in both DM and non-DM patients with STEMI, whereas glucose level was the best predictor in patients with NSTEMI. Yang et al. [8] enrolled 4,362 coronary artery disease patients with a study endpoint of MACCEs and a median follow-up period of 2.5 years and showed that the SHR is a useful predictor of MACCEs after PCI, especially in non-DM patients with STEMI. To the best of our knowledge, the current study may be the first to show a dose–response relationship between the SHR and IHCA in patients with ACS treated with PCI. In our study, we included 1,939 patients with ACS treated with PCI. The results showed that SHR was significantly associated with the incidence of IHCA in patients with ACS (OR = 2.6800; 95% CI = 1.6200–4.4300; *p* < 0.001) and the OR of IHCA was significantly increased when the SHR was > 1.773, even after adjusting for confounders.  Further RCS analysis showed that this correlation was a dose-response relationship. In addition, baseline data showed that DM incidence and insulin or glucose-lowering medication use were higher in the T3 group than in the T1 group. We hypothesised that patients in the T3 group might experience more hyperglycaemic episodes due to the inappropriate use of insulin or glucose-lowering medication, which has been shown to increase the risk of cardiovascular events [40, 41]. *P*-values for all interactions were > 0.05 in the subgroup analyses, and the different results in the subgroup analyses may be due to an insufficient sample size. However, the underlying mechanisms by which the SHR shows a dose–response relationship with IHCA in patients with ACS remain uncertain and may include the following. The duration of hyperglycaemia appears to be critical in determining whether hyperglycaemia is protective or harmful, that is, long-term hyperglycaemia is harmful, whereas short-term hyperglycaemia is beneficial [42, 43]. Hyperglycaemia in the context of acute disease is an evolutionarily conserved adaptive response that increases the host's chances of survival [44]. Hyperglycaemia can trigger compensatory mechanisms that offer protection against ischemia and potentially guard against post-ischemic cell death by promoting anti-apoptotic and cell survival pathways and angiogenesis [45, 46].  Thus, when SHR is <1.773, the mild-to-moderate SHR in this study may be protective against IHCA events. In our study, SHR >1.773 may be a true SIH. When the SHR is < 1.773, it indicates chronic hyperglycaemia (high HbA1c), with either good current glycaemic control or overcontrol (low ABG). Hence, the curve's steepness will be higher for outcomes closely linked to acute responses, while the curve will be relatively flat for outcomes more connected to chronic hyperglycaemia. In the future, larger prospective cohort studies should be conducted to determine the SHR threshold for the diagnosis of SIH and to explore its predictive value for cardiovascular outcomes in patients with ACS. In conclusion, although there was no interaction of SHR on IHCA between the DM and non-DM subgroups (*p* = 0.7211), the dose–response relationship between the SHR and IHCA may be partly due to DM status.

### Strengths and limitations

To the best of our knowledge, this is the first time a dose–response relationship between the SHR and IHCA in patients with ACS has been proposed using RCS analysis, and the linear correlation between the SHR and ICHA events was analysed and evaluated. However, this study has some shortcomings. First, it was a single-centre study that included only Asian patients, and these results should be interpreted with caution. Second, the current study is limited by its retrospective design, and causality cannot be inferred; further prospective multicentre studies are needed to validate these results. In addition, we cannot exclude the possibility of unmeasured or unknown confounding factors that may explain the associations observed in this study.

## Conclusions

In patients with ACS treated with PCI, the SHR was significantly associated with the incidence of IHCA. The SHR may be a valid predictor of the incidence of IHCA in patients with ACS treated with PCI. In addition, the inclusion of the SHR in the baseline risk model had an incremental effect on the predictive value of IHCA in these patients. More prospective, large-scale, multicentre studies should be conducted to assess the predictive value of the SHR in patients with ACS; the potential mechanism of the dose–response relationship requires further study.

## Data Availability

Due to privacy and ethical constraints, the datasets generated and analysed in this study are not publicly available but can be obtained from the corresponding author.

## Abbreviations

SHR: Stress hyperglycaemic ratio
DM: Diabetes mellitus
PCI: Percutaneous coronary intervention
STEMI: ST-elevation myocardial infarction
NSTEMI: Non-ST-elevation myocardial infarction
UA: Unstable angina
TG: Triglycerides
ABG: Admission Blood Glucose
HbA1c: Haemoglobin A1c
EGFR: Estimated glomerular filtration rate
SIH: Stress-induced hyperglycaemia
ACS: Acute coronary syndrome
IHCA: In-hospital cardiac arrest
EGFR: Estimated glomerular filtration rate
TG: Triglycerides
Cr: Creatinine
OR: Odds ratio
CI: Confidence Interval
LASSO: Least absolute shrinkage and selection operator
ROC: Receiver operating characteristic
AUC: Area under the curve
NRI: Net reclassification index
IDI: Integrated discriminant improvement index
AMI: Acute myocardial infarction
MACE: Major adverse cardiovascular event
MACCE: Major adverse cardiovascular and cerebrovascular event
